# COVID-19 surveillance in wastewater: An epidemiological tool for the monitoring of SARS-CoV-2

**DOI:** 10.3389/fcimb.2022.978643

**Published:** 2023-01-05

**Authors:** Sajida Maryam, Ihtisham Ul Haq, Galal Yahya, Mehboob Ul Haq, Abdelazeem M. Algammal, Sameh Saber, Simona Cavalu

**Affiliations:** ^1^ Department of Biosciences, The Commission on Science and Technology for Sustainable Development in the South (COMSATS) University Islamabad (CUI), Islamabad, Pakistan; ^2^ Department of Physical Chemistry and Polymers Technology, Silesian University of Technology, Gliwice, Poland; ^3^ Joint Doctoral School, Silesian University of Technology, Gliwice, Poland; ^4^ Department of Microbiology and Immunology, Faculty of Pharmacy, Zagazig University, Zagazig, Egypt; ^5^ Department of Bacteriology, Immunology, and Mycology, Faculty of Veterinary Medicine, Suez Canal University, Ismailia, Egypt; ^6^ Department of Pharmacology, Faculty of Pharmacy, Delta University for Science and Technology, Gamasa, Egypt; ^7^ Faculty of Medicine and Pharmacy, University of Oradea, Oradea, Romania

**Keywords:** wastewater surveillance, point-of-care diagnostics, COVID-19 pandemic, COVID-19 surveillance system, early warning system

## Abstract

The coronavirus disease 2019 (COVID-19) pandemic has prompted a lot of questions globally regarding the range of information about the virus’s possible routes of transmission, diagnostics, and therapeutic tools. Worldwide studies have pointed out the importance of monitoring and early surveillance techniques based on the identification of viral RNA in wastewater. These studies indicated the presence of the severe acute respiratory syndrome coronavirus 2 (SARS-CoV-2) RNA in human feces, which is shed *via* excreta including mucus, feces, saliva, and sputum. Subsequently, they get dumped into wastewater, and their presence in wastewater provides a possibility of using it as a tool to help prevent and eradicate the virus. Its monitoring is still done in many regions worldwide and serves as an early “warning signal”; however, a lot of limitations of wastewater surveillance have also been identified.

## Introduction

Economic stability and the security of human health are considerably affected by infectious diseases as they cause one-fourth of the mortalities around the world. Even with the development of advanced healthcare systems, viral infections still continue to emerge (Bloom and Cadarette, 2019). The recent outbreak of coronavirus disease 2019 (COVID-19) appeared to be highly pathogenic and life-threatening, with high mortality rates, affecting almost every country in the world (Ming, 2020). The symptoms include fatigue, fever, dry cough, muscle pain, and shortness of breath (Chen et al., 2020; Huang et al., 2020; Muhammad et al., 2021; Egbuna et al., 2022). Initially, the disease was believed to be pneumonia by healthcare officials; however, a thorough analysis of the patient’s throat sample detected a novel coronavirus (CoV) (Ulhaq et al., 2021). On January 7, another CoV pathogen in humans was discovered; provisionally, it was named novel coronavirus 2019 (nCoV-19) by the World Health Organization (WHO). Because of its genetic similarity with severe acute respiratory syndrome coronavirus (SARS-CoV), it was then recognized as SARS-CoV-2 (severe acute respiratory syndrome coronavirus 2) according to the viral nomenclature scheme (Basit et al., 2021). The disease caused by SARS-CoV-2 was initially named viral pneumonia; the WHO later gave it the name coronavirus disease 2019 (COVID-19) due to the presence of a number of unique disease symptoms of the lower respiratory tract, such as difficulty in breathing (WHO 2019-nCoV Situation Report-22). Moreover, patients also suffer from mental dissatisfaction, acute kidney failure, and dysfunction of various other organs in extreme cases of COVID-19 (Basit et al., 2021). However, the incubation period fluctuates based on the patient’s health status and immunity, where it can be shorter in older people and in those with a weak immune system. COVID-19 is rapidly transmitted through respiratory droplets due to its more contagious characteristic (Jiang et al., 2020). More than 213 countries have reported over 40 million confirmed cases, with nationwide lockdowns imposed to prevent the spread of the disease (Kataki et al., 2021).

SARS-CoV-2 had been detected in the blood and in anal and oropharyngeal swabs, along with urine samples of patients who tested positive (Chen et al., 2020; Ren et al., 2020), indicating the presence of this deadly viral RNA in the streamlets of wastewater treatment plants (WWTPs) (Foladori et al., 2020). The swab protocols are mostly used for the collection of target pathogens from the environment and from patients (Colaneri et al., 2020; Moore et al., 2021), but with various disadvantages. These include the inability to perform collection from living environments and the possibility of getting false-positive signals due to the free RNA fragments of lysed viruses from the environment and patients, as they can remain stable for long periods of time. The European Union (EU) Commission suggested a systematic surveillance approach for SARS-CoV-2 in EU wastewaters (Commission, E, 2021). Patients infected with SARS-CoV-2 do not always have the typical symptoms (Nishiura et al., 2020), as almost 40%–50% of infections do not show any symptoms (Oran and Topol, 2020), but can still spread the virus (Gandhi et al., 2020). Both asymptomatic and symptomatic individuals shed SARS-CoV-2 in the urine, saliva, feces, and nasal fluids (Cevik et al., 2021). The analysis of fecal samples is more sensitive for the detection of SARS-COV-2 as feces contain a higher viral load (Yuan et al., 2021). Reports showed that the presence of SARS-CoV-2 RNA in human feces (Holshue et al., 2020; Song et al., 2020) and urine leads to the shedding of the viral RNA in wastewater (Ding et al., 2020; Gao et al., 2020; Holshue et al., 2020; Jiehao et al., 2020; Wang et al., 2020; Woelfel et al., 2020). Several studies have identified the viral RNA in wastewater (Ahmed et al., 2020a; Medema et al., 2020). COVID-19 consequently causes alarming situations worldwide, posing a cluster of questions for the scientific community regarding the contemporaneous exploration of epidemiological studies such as wastewater surveillance. Therefore, methodologies need to be developed using wastewater for epidemiological studies of human diseases such as COVID-19.

The epidemiology of human viruses in wastewater can be beneficial for population-based analysis of the epidemiology of SARS-CoV-2 to prevent its further transmission (Hillary et al., 2021). Viral detection of CoVs in wastewater was first developed in 2013 (Wong et al., 2013). The genome of the human coronavirus HKU1 (HCoV-HKU1) was identified in sewage (Bibby and Peccia, 2013), and animal CoV had also been found in surface water (Blanco et al., 2019). Environmental surveillance (ES) based on wastewater sampling enables the prediction of the condition of drainage areas with little effort compared to conducting clinical surveys (Agrawal et al., 2021). Pre-symptomatic and asymptomatic infected individuals normally are not included in clinical surveys. Sample collection and testing are usually expensive and time-consuming. A lot of studies have shown that the monitoring of wastewater can detect the outbreak of viruses such as norovirus and poliovirus faster than clinical surveys (Hovi et al., 2012; Hellmér et al., 2014; Daughton, 2020; Hata and Honda, 2020). Its potential advantage is the possibility to conduct screening of a large population using a few samples without depending on the availability of clinical testing (Thompson et al., 2020), with transmission collections in large geographic regions that can assist in rapid efforts by public health authorities (Stadler et al., 2020).

## Evidence of viral RNA in fecal material

Coronavirus get into fecal material through the swallowing of secretions from the upper respiratory tract, which is then mixed with food when it is not affected by gastric acid. Viral replication in intestinal cells or infected immune cells plays a role in the presence of CoV in feces (Ding et al., 2020; Gu et al., 2020). Its replication in intestinal samples had been identified, determining the gastrointestinal tract (GIT) of humans as a site of SARS-CoV-2 infection (Ding et al., 2004). SARS-Cov-2 replication can be confirmed by the presence of the viral RNA in feces (Wang et al., 2020). A study showed that the rectal mucosa is altered by infection with SARS-CoV-2 and exhibited a replication. The virus was replicated in the rectum during the period of incubation, while the viral particles showed in the epithelial cells of the patient’s intestine (Qian et al., 2021). Occasionally, urine samples of infected individuals contain viral RNA, but the swabs from the throat result as negative (Xu et al., 2020). The SARS-CoV-2 RNA has been reported earlier from throat and nasal swabs compared to fecal samples (Zhang et al., 2020c). A lot of studies have shown this fact, as in one case that revealed the SARS-CoV-2 RNA being contained in throat and nasal swabs (TS and NS, respectively) for only 9 days after ailment, while the fecal RNA remained for more than 20 days (Cai et al., 2020). Another report showed the viral RNA in fecal material for up to 4–5 weeks (Cai et al., 2020; Zou et al., 2020). Generally, infected patients are discharged from hospital when the NS and TS samples are negative, even if the fecal samples are still positive for SARS-CoV-2 RNA; consequently, the oral–fecal route implication needs to be highlighted, particularly in the context of the stability of the SARS-CoV-2 virus in sewage water. Moreover, its fecal shedding recommends demonstrating the decontamination practices for toilets and the observation of hygiene and sanitation practices (Parasa et al., 2020).

## Gastrointestinal symptoms of COVID-19 and shedding of SARS-CoV-2 in excreta

The presence of *ACE2* in the intestinal cells confirms the entry of SARS-CoV-2 in the GIT, with the presence of SARS-CoV-2 in the stool and diarrhea having been reported (Muhammad et al., 2021). Examination of the esophagus revealed lymphocyte intrusion in the squamous epithelium, stomach lamina propria, duodenum, and interstitial edema, along with various gastrointestinal symptoms of COVID-19 such as nausea, vomiting, and diarrhea (Leung et al., 2003; Memish et al., 2020). These symptoms were shown by nearly 2%–10% of patients (Wang et al., 2005; Gao et al., 2020; Wang et al., 2020). The virus usually infects epithelial cells of the GIT (Ding et al., 2020); moreover, the RNA of SARS-CoV2 has been detected in 40%–85% of fecal samples, indicating a similar frequency of the detection of SARS-CoV-2 RNA in feces to respiratory secretions (Natarajan et al., 2022). According to Ling et al. (2020), 81.8% of studies on CoV reported the detection of SARS-CoV-2 in stool samples. The viral load is nearly 108 copies/g of feces (Lescure et al., 2020; Pan et al., 2020; Woelfel et al., 2020), 107 copies/ml when there is diarrhea, and 2.5 × 104 copies/ml in the case of urine (Hung et al., 2004). Viral shedding in fecal material continues for almost 7 weeks after the onset of the first symptom (Ding et al., 2020; Jiehao et al., 2020). Globally, there are various reports on the replication and infection of SARS-CoV-2 in the GIT, as shown in **Table 1**. With the rapid growth of the global population, the emergence and reemergence of human pathogenic viruses have prompted a demand for surveillance systems that understand the dynamics of infection in populations (Mao et al., 2020). The widespread circulation of SARS-CoV-2 variants calls for wastewater-based epidemiology to examine its magnitude and distribution in a community by examining its biomarker levels in a sewage network. Detection of the presence of SARS-CoV-2 in wastewater can be utilized as a disease surveillance tool, which has been supported worldwide by many researchers (Ahmed et al., 2020a; Lodder and de Roda Husman, 2020).

**Table 1:** Worldwide studies on the replication and infection of SARS-COV-2 in stool and fecal samples.

Virus	Sample	No. of patients	Location	Symptoms	Reference
Live SARS-CoV-2	Stool	2	China	No diarrhea	Wang et al., 2020
Cultivation of SARS-CoV-2	Single stool specimen	1	China	Severe pneumonia	Zhang et al., 2020b
Viral RNA	Feces	Multiple	China	Infected gastrointestinal cells	Ding et al., 2020
SARS-CoV-2 RNA	Fecal	113	Nguyen	Abdominal pain, vomiting, and nausea	Natarajan et al., 2022
SARS-CoV-2 RNA	Fecal	22	China	Fever and respiratory symptoms	Zhang et al., 2020d
2019-nCoV RNA	Fecal	14	China	Severe symptomatic stage	Zhang et al., 2020e
Viral RNA	Fecal	28	China	Gastrointestinal symptoms	Udugama et al., 2020
Viral RNA	Fecal	9	Hongkong	Gastrointestinal symptoms (nausea, loss of appetite, diarrhea, vomiting, and abdominal pain or discomfort)	Cheung et al., 2020
Viral RNA	Stool swab	1	China	Mild fever and diarrhea	Cho and Ha 2020
SARS-CoV-2 RNA	Fecal	3	UK	COVID positive symptoms	Vaselli et al., 2021
SARS-CoV-2 RNA	Stool	7	USA	Mild to moderately severe illness	Kujawski et al., 2020
SARS-CoV-2 RNA	Loose bowel movement	1	USA	Abdominal discomfort, nausea and vomiting	Holshue et al., 2020
SARS-CoV-2 RNA	Anal swabs	4	China	COVID symptoms + admission to hospital	Zhang et al., 2020c
SARS-CoV-2 RNA	Stool samples	93	China	With critical, severe, moderate, and mild symptoms	Zhang Y. et al., 2020
SARS-CoV-2 RNA	Stool	212	China	Pediatric patients with SARS-CoV-2 infection	Yuan et al., 2020
SARS-CoV-2 RNA	Stool	4	Singapore	Mild respiratory tract infection	Young et al., 2020
SARS-CoV-2 RNA	Stool	3	China	Mild to moderate severity and fever	Xing et al., 2020
SARS-Cov-2 RNA	Stool	2	Germany	One is asymptomatic, one is symptomatic	Hoehl et al., 2021

SARS-CoV-2, severe acute respiratory syndrome coronavirus 2; nCoV, novel coronavirus.

The SARS-CoV-2 RNA has been reported in groundwater, surface water, wastewater, sludge, and other hospital-related water systems during the low- and middle-risk periods of COVID-19 (Zhao et al., 2020). The concentration and the molecular detection of SARS-CoV-2 RNA in wastewater isolated from different localities throughout the world are shown in **Table 2**. The necessity for surface hygiene can be explained by the detection SARS-CoV-2 in the toilets of infected patients (Ding et al., 2020). Moreover, SARS-CoV-2 was also found in natural waters derived from areas with poor sanitation, which has serious consequences on health and the environment (Guerrero-Latorre et al., 2020). However, RNA detection in wastewater lacks an appropriate standard protocol. The method used to calculate the concentration of viruses is not that effective for the recovery of these viruses (Haramoto et al., 2009; Ye et al., 2016).

**Table 2:** Details of the reported molecular detection of severe acute respiratory syndrome coronavirus 2 (SARS-CoV-2) in wastewater.

Sampling location	Water type	Virus detection method	Sequence confirmation	Detection results: positive rate and maximum concentration (copies/L)	Reference
Australia	Untreated wastewater	Electronegative membrane-direct RNA extraction; ultrafiltration	Direct sequence of qPCR products (Sanger+MiSeq)	22% and 1.2 × 10^2^	Ahmed et al., 2020a
Netherlands	Untreated wastewater	Ultrafiltration	Not done	58% not available	Medema et al., 2020
USA	Untreated wastewater	PEG precipitation	Direct sequencing of qPCR products (Sanger)	71% and >2 × 10^5^	Wu et al., 2020
France	Untreated water	Ultracentrifugation	Not done	100% and >106.5	Wurtzer et al., 2020a
USA	Untreated wastewater	Ultrafiltration	Re-amplification by regular PCR followed by Sanger sequencing	100% and >3 × 10^4^	Nemudryi et al., 2020
Italy	Sewage	PEG–dextran method	Nested RT-PCR assays and one real-time qPCR assay	50% (6/12) shown	La Rosa et al., 2020

PEG, polyethylene glycol; qPCR, quantitative polymerase chain reaction.

## Approaches used in wastewater-based epidemiology

Surveillance provides insights into the condition of the outbreak in an area by testing the wastewater samples with time. The surveillance data are used in three ways: to monitor the presence of infections in a community, to track the infection trends in a community, and to perform targeted screening of the infections for moderation measures (CDC, 2022). There are various types of wastewater-based epidemiology (WBE), which require research for validation. The qualitative approach examines the minimum level of infection, which detects sensitivity, the semi-qualitative approach can indicate the comparative level of infection in a community, while the quantitative approach can detect the absolute infection level and is capable of performing comparisons across communities. Initially, these approaches were used as tools to assess illegal drug use within a community (Daughton, 2001) by quantifying them with specific human metabolites in wastewater (Feng et al., 2018). Sewage RNA collection can both indicate past and active infections when considering the duration of RNA excretion through the stool (Chen et al., 2020; Wu et al., 2020). Viral shedding can occur right after infection; however, an infected patient being identified through this system must show symptoms and start treatments before clinical diagnosis (Larsen and Wigginton, 2020). Surveillance *via* wastewater can be used as an indicator of disease transmission and its ratios increasing and decreasing, such as, traditionally, deaths, hospitalizations, serological data, and test positivity (Rowe et al., 2009). Similarly, daily surveillance of SARS-CoV-2 RNA in wastewater can provide evidence, similar to daily individual testing in a community, but with a less invasive and cost-effective method reconfirming the already diagnosed cases. Population size is important in the use of the quantitative approach, which can determine the infected population (Daughton, 2012; Daughton, 2018). Calibration of the monitoring of WBE is important so that the data will be related to the infection rates, which can be conducted utilizing quantitative PCR (qPCR) with reverse regression of the known rates of infection (Bar-Or et al., 2021). This will help in the estimation of the total number of infected people, reliably enabling a comparison of the infection rates in communities. Moreover, the monitoring information should be presented and interpreted to the public for awareness; experts and scientific authors can play an important role. The involvement of policy makers, government health officials, and leaders would also help in this regard (Daughton, 2020).

### SARS-CoV-2 surveillance in wastewater

The wastewater surveillance approach is presently utilized all over the world as an effective tool for SARS-CoV-2 RNA monitoring (Bivins et al., 2020). It is not only limited to the detection of COVID-19, but was also previously utilized for viruses such as hepatitis A and poliovirus (Asghar et al., 2014; La Rosa et al., 2014; Bivins et al., 2020). This surveillance method is currently utilized in almost 55 countries across the world with a declaration from over 250 organizations to analyze wastewater samples for SARS-CoV-2 RNA from more than 2,690 sites, including surface waters and WWTPs (Naughton et al., 2021). Some of these observations were done even before the appearance of the first positive case clinically (Medema et al., 2020). Therefore, this surveillance technique is useful in indicating the presentation of SARS-COV-2 and other viruses in communities along with the estimation of its effectiveness in the healthcare field (Kitajima et al., 2020). Wastewater can be a factor in the outbreak of SARS viruses because of improper sewage systems (McKinney et al., 2006). Retrospectively, in 2004, wastewater epidemiology was used for SARS-CoV, which found that almost 30% of disinfected wastewater and 100% of untreated water contained the virus during the first outbreak in China (Wang et al., 2005). The prevalence of SARS-CoV-2 was determined by detecting the RNA copies of the virus in the Australian sewage basin using reverse transcription quantitative PCR (RT-qPCR). This surveillance method was also used in the USA to study the strains of SARS-CoV-2, their phylogeny, ancestry, and the effectiveness of interventions for public health in reference to the outbreak (Nemudryi et al., 2020). Wastewater surveillance for COVID-19 around the world is shown in **Table 3**.

**Table 3:** Worldwide studies on wastewater surveillance.

Sample	Location	Technique used	Reference
Raw wastewater samples	France	RT-qPCR	Wurtzer et al., 2020a
3 sewage samples	China	RT-qPCR	Wang et al., 2020
Wastewater treatment plant	France	RT-qPCR	Trottier et al., 2020
Wastewater samples	Pakistan	RT-qPCR	Sharif et al., 2020
Primary sewage sludge	US metropolitan area	RT-qPCR	Peccia et al., 2020
Wastewater samples	Canada	RT-qPCR assay	Neault et al., 2020
Sewage samples	Netherlands	RT-PCR	Medema et al., 2020
Untreated wastewater	Italy	SARS-CoV-2 RNA	La Rosa et al., 2020
Raw water samples at a pumping station	Argentina	RT-qPCR	Iglesias et al., 2021
Influent wastewater samples	Japan	Several PCR-based assays	Hata and Honda, 2020
Wastewater of sewage systems	Spain	RT-PCR	Fernández-de-Mera et al., 2021
Human sewage	Brazil	RT-qPCR	Fongaro et al., 2021
Wastewater treatment plants	India	RT-qPCR	Arora et al., 2022
Treated and untreated wastewater	Chile	RT-qPCR	Ampuero et al., 2020
Wastewater	Australia	RT-qPCR	Ahmed et al., 2020a
Sewage sludge	USA	RT-qPCR	Peccia et al., 2020

SARS-CoV-2, severe acute respiratory syndrome coronavirus 2; RT-qPCR, reverse transcription quantitative polymerase chain reaction.

In Australia, wastewater surveillance detected the SARS-CoV-2 RNA using sequencing and qPCR (Agrawal et al., 2021); however, further studies are needed to understand the reliability of the analysis. A lot of studies have described the detection of SARS-CoV-2 within sewage or wastewater in USA, Australia, France, and Netherlands (Lodder and de Roda Husman, 2020; Medema et al., 2020; Nemudryi et al., 2020; Wu et al., 2020; Wurtzer et al., 2020b). Most of these studies were done without treatments and had a maximum concentration of 106 copies/L (Wurtzer et al., 2020a). These studies, along with several other continuous efforts in different regions of the world, were conducted to provide updates on the community prevalence of SARS-CoV-2 and its epidemiology. This surveillance method can also be used as a “warning signal” to alleviate the spread of infection in communities, as an outline describing the ethical issues in relation to the basic approach to sanitation (Murakami et al., 2020).

ES can help provide information on the transmission of infection within a community before clinical surveillance is done. This serves as an early warning system, as the shedding of SARS-CoV-2 begins even before the onset of symptoms in an infected person (Jones et al., 2020). It can also highlight the underreported characteristics from clinical surveillance due to several testing policies; thus, ES can assist in better monitoring of the incidences of COVID-19 (Choi et al., 2018). It can also be beneficial in international airports, voyage ships, and aircraft for monitoring the SARS-CoV-2 drifts among travelers (Medema et al., 2020). Analysis at the molecular level in a community can paint a picture of the existing and emerging variants of the virus (Izquierdo-Lara et al., 2021). An alarming fact is that, in many poor countries, the virus is transported to treatment plants for the dumping of human waste, which accounts for almost 900 million public globally (WHO, 2017). Underground water or soil can also become contaminated due to lack of sanitation. A number of previous outbreaks also showed viral shedding *via* human excreta (Goh et al., 2013; Zhou et al., 2017; Yeo et al., 2020). The consequences of COVID-19 indication in wastewater were given by Nghiem et al. (2020), with a description of the fecal–oral transmission routes by Heller et al. (2020).

#### Understanding COVID-19 epidemiology through wastewater surveillance

The presence of the viral RNA of SARS-CoV-2 in wastewater creates the possibility of employing it as a tool to study viral genomics, epidemiology, and prevalence, as well as possible eradication from the community (Kitajima et al., 2020). The transmission routes of SARS-CoV-2 include seepage wastewater as reusable water, biosolid products from sludge, thrusting, mixing, and microbial vaporizer. Viruses need to be viable to cause infection through these systems. According to updated information, the viability of CoVs decreases in wastewater, and their infectivity should also be diminished upon transfer from feces to effluent and then to treatment plants and the environment (La Rosa et al., 2020). The epidemiology of wastewater can be an important method to trace the viral circulation in a community in order to evaluate the prevalence and genomic diversity (Sinclair et al., 2008; Xagoraraki and O’Brien, 2020).

**Figure 1:**
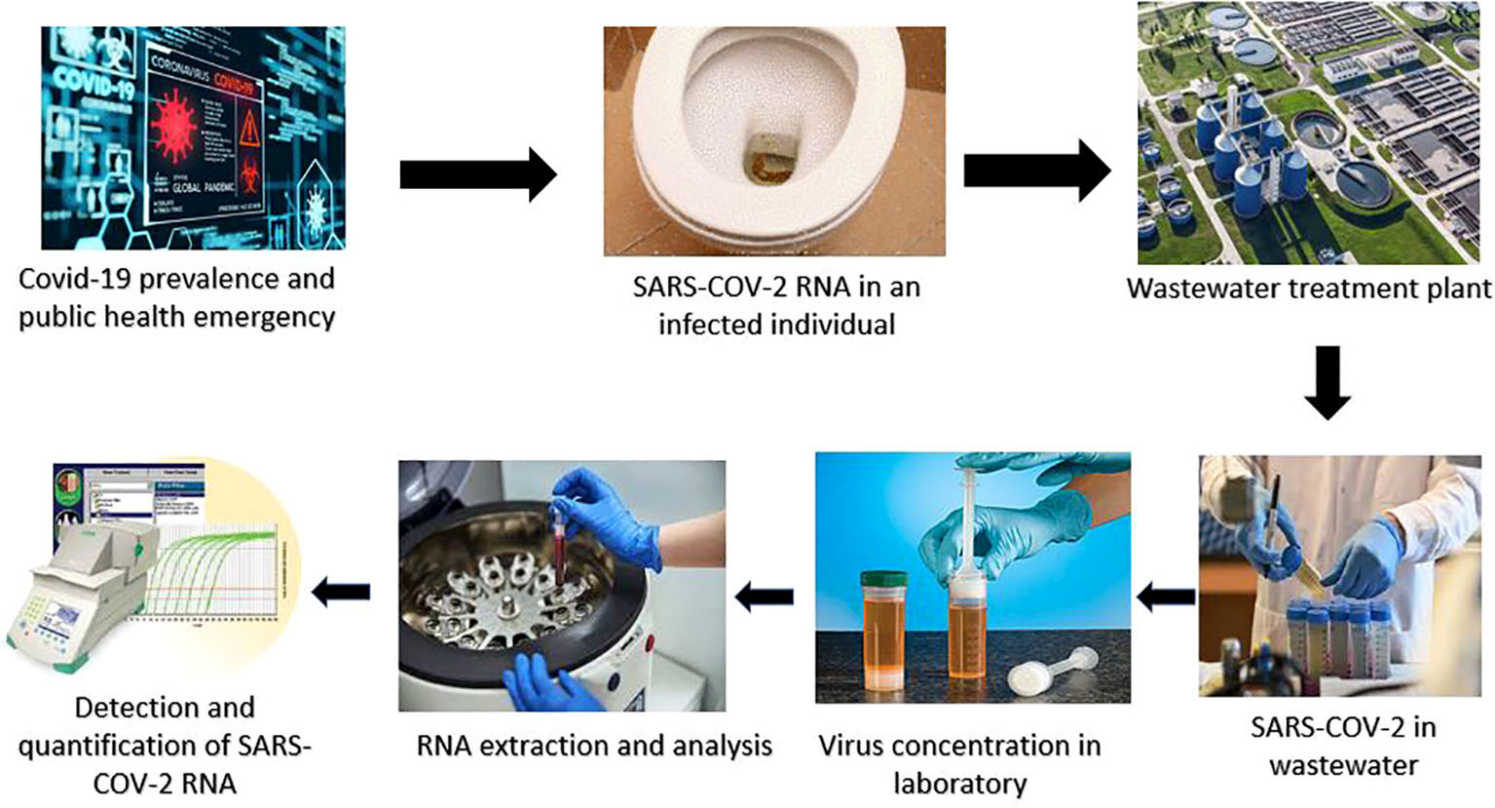
Steps of wastewater-based epidemiology (WBE) by testing wastewater to monitor the outbreak of SARS-CoV-2..

Wastewater systems can provide an opportunity to detect the virus extracted from feces (Carducci et al., 2006; La Rosa and Muscillo, 2013). With this method, monitoring the epidemiology of the virus has become possible, with the traditional techniques being somehow limited due to viral infections that do not cause any symptoms (Johansson et al., 2014; Qi et al., 2018). **Figure 1** shows the surveillance process related to sludge and wastewater.

It is rather difficult to determine the circulation of a virus in a community through comparisons between regions with varied capabilities of viral diagnosis (Ortiz-Ospina and Hasell, 2020). Viral surveillance is an impartial method of evaluating the viral spread in different regions, even when there is a limitation in resources and diagnosis. Monitoring can also enable the detection of changes in the viral strains with genomic analysis from an evolution perspective (Lodder et al., 2013; La Rosa et al., 2014).

Wastewater surveillance can detect viruses even at low levels, such as when there is a decrease in cases due to healthcare interventions (Asghar et al., 2014). Moreover, it is important when a novel virus or strain is introduced into a population (Sinclair et al., 2008; Savolainen-Kopra et al., 2011) due to seasonal changes or fluctuations (Sedmak et al., 2003; Hellmér et al., 2014; Prevost et al., 2015). Therefore, these strategies can be useful as an “early warning” system (Xagoraraki and O’Brien, 2020) to determine any alterations in public health involvement, as in the case of social isolation and lockdowns with the recurrence of SARS-CoV-2. A lot of preventive measures can be implemented by performing wastewater analysis for viruses and by being able to detect any novel viruses earlier than clinical detection (Ng et al., 2012; Fernandez-Cassi et al., 2018; Bibby et al., 2019). The total number of affected individuals can be estimated by detecting the RNA copy numbers of SARS-CoV-2 in untreated wastewater *via* catchment site and by using the Monte Carlo simulation. This statistical method is employed to resolve complex issues and determine possible outcomes using estimates (Sin and Espuña, 2020), which is a simple and easy-to-use concept. The estimation of almost 1,090 infected patients with clinical observations was conducted using this method (Ahmed et al., 2020a). Studies have pointed out the need for more strategies and molecular-based assays to validate the presence of viruses in water, which will increase the accuracy of wastewater surveillance.

#### Limitations in wastewater surveillance

In diagnostics, various factors can affect the reliability of the results (Bustin et al., 2009; Medema et al., 2020; Pecson et al., 2021). False-positive errors can result from high sensitivity developed *via* the analysis of wastewater samples using poor RT-PCR assays (Ahmed et al., 2022). The application of WBE can be a bit difficult due to poor sanitation and the lack of treatment plants for wastewater (Ahmed et al., 2021; Jakariya et al., 2021). Therefore, currently, it is not likely to entirely alter the viral RNA levels in wastewater for its prevalence (Larsen and Wigginton, 2020). Several limitations of wastewater surveillance have been identified, such as in relating the identified infected cases and the virus levels. As viral excretion can be altered during the infection period, delays can occur due to different time frames, capturing altered population distributions due to traveling, and fluid dilution from precipitation, among others (La Rosa et al., 2020). Moreover, the risk of genome stability in wastewater, alterations in the sampling type, poor efficiency of the viral concentration technique, and unavailability of a sensitive screening assay can limit the quantification of viral detection, especially when the concentration is low. The pandemic can become worse when appropriate testing trials fail to efficiently determine patients infected with SARS-CoV-2 (Mercer and Salit, 2021). Additionally, these patients are extremely contagious either at the pre-asymptomatic or the asymptomatic stage. Hence, without a proper screening assay, they can infect healthy individuals earlier than they can be identified for isolation or hospitalization (Oran and Topol, 2020). Consequently, for the accurate detection of COVID-19, new molecular-based methods have been recently developed. These include a microarray-based method, loop-mediated isothermal amplification (LAMP) method, RNA targeting CRISPR (clustered regularly interspaced short palindromic repeats) diagnosis, rolling circle amplification-based method, and nanopore targeted sequencing (NTS) (Basit et al., 2021).

Some of these diagnostic methods have not been independently assessed or approved by authorities of healthcare systems. The iAMP COVID-19 detection kit (Atila BioSystems, Mountain View, CA, USA) based on isothermal amplification (LAMP) technology has been approved by the U.S. Food and Drug Administration (FDA) with 100% sensitivity (BioSystems, A, 2020). Another kit, Sherlock™ CRISPR (Sherlock Biosciences, Boston, MA, USA), which is based on CRISPR technology, can detect SARS-CoV-2 with a specificity of 100% (Biosciences, S, 2020), while the microarray method allows the detection of various microbial agents through the amplification of nucleic acids with high specificity (Vora et al., 2004) and previously showed results of the detection of CoVs (Shi et al., 2003). The limit of detection and the clinical sensitivity are considered crucial criteria for commercial molecular diagnostics in the case of COVID-19 diagnosis (Bachelet, 2020; Tang et al., 2020). Currently, there are only two kinds of commercial diagnostic tests: serological tests to detect CoV-2 antibodies in serum and molecular diagnostics to determine viral RNA in respiratory specimens (Sheridan, 2020). The next-generation sequencing-based method Swab-Seq is another technique utilizing RT-PCR primers, which has exclusive molecular codes for the sequencing of many samples (Bloom et al., 2020). Decentralized wastewater samples are quite difficult to manage due to the rigorous sample collection process for households (Takeda et al., 2021). Analysis of the high-risk points is needed as a solution to this problem, which includes quarantine areas, hospitals, and healthcare services. Furthermore, samples from sludge plants (collected from decentralized systems) should be collected (UNDP, 2020). Various shareholders need to be involved in the processes of a monitoring framework, including sampling, analysis, information feedback, and any subsequent action or decision-making. Sample analysis would require equipment for the health and research sectors involving national and international platforms for wastewater surveillance (UN Women, 2020). Budgets should be established for prevention and response, along with the establishment of committees to meet regional and international goals (Takeda et al., 2021). The calibration of viral RNA needs to be performed against the exact number of cases presented by marking and targeting biomarkers (Daughton, 2020). The future of wastewater surveillance needs to be assessed more in light of vigorous epidemiological data, as enormous limitations of testing potential have been observed over time, especially in communities where delays in testing can occur.

### SARS-CoV-2 diagnosis from wastewater samples

The presence of SARS-CoV-2 in wastewater samples can be confirmed by determining the viral RNA sequence with qPCR (WHO, 2020). In this regard, the positive control can be shown by determining the plasmid with the complete nucleocapsid gene of SARS-CoV-2 (Wu et al., 2020). Different pretreatment techniques are usually performed during the sampling of wastewater to ensure efficient detection with maximum viral concentration. A lot of approaches for the enrichment of different viruses have been recently used, including PCR assays; however, there is still a lack of a standard protocol. The initial diagnostic test was based on the specific primers and sequences for PCR. This test was developed based on previous research works on the detection of SARS-CoV-2 (Udugama et al., 2020). Several companies have started commercializing them for a faster supply of tests (Vandenberg et al., 2021).

The active sampling approach has been demonstrated for the quantification of SARS-CoV-2 in wastewater, along with the trends of infection (Graham et al., 2020; Castro-Gutierrez et al., 2022), with several methods to determine concentrations having been advised for wastewater (Rusiñol et al., 2020; Barril et al., 2021; Cervantes-Aviles et al., 2021; Chik et al., 2021; Philo et al., 2021). Most of these focused on extrinsically sourced viral controls in water (Kantor et al., 2021). Filtration, nuclease treatment, and freeze–thaw methods are among the reputable and rapid procedures for viral RNA separation from host RNA (Marston et al., 2013; Dupinay et al., 2014; Hall et al., 2014). The main aim is to avoid the reduction of the overall viral RNA quantity during separation from the host RNA (Victoria et al., 2008; Hall et al., 2014). In this regard, microfluidic devices are far more dependable compared to the traditional methods because of their requirement of less volume of biological samples for disease biomarker testing within a short time. Moreover, parallel analysis assays based on a single microfluidic device can provide the best statistical results (Verpoorte and De Rooij, 2003; Zhang et al., 2013). The gold standard for SARS-CoV-2 diagnostic is RT-PCR (Carter et al., 2020), which is specific, reliable, and sensitive. Numerous costly instruments and highly trained professionals are needed to perform the tests, which is a concern in developing countries (Afzal, 2020). On the contrary, the RT-LAMP test reduces the detection time to almost 30 min, enabling rapid detection (Lamb et al., 2020). The available microfluidic kits offer a rapid, cost-effective, and precise detection overall; therefore, they are appropriate in settings where resources are scarce for point-of-care testing (POCT). Integrated microfluidic devices have applications in whole-genome sequencing, COVID-19 progression, and intratracheal neutralization of a virus (Jamiruddin et al., 2022).

#### Polymerase chain reaction

In molecular biology, PCR is the quick and broadly used method to make DNA copies from thousands to millions containing the fragment of a gene. A very small quantity of DNA (genetic material) can be amplified by PCR and provide adequate evidence of the DNA or gene segment for comprehensive study. Most viral diseases are diagnosed using PCR due to its broader impact and reliability, indicating its value for routine use in the diagnosis of various infections such as COVID-19 (Basit et al., 2021). The etiological agent of COVID-19 is SARS-CoV-2, which is an RNA virus that can be converted to complementary DNA (cDNA) by the process of reverse transcription through the reverse transcriptase enzyme. Subsequently, with the use of specific primers, the DNA sample is amplified by PCR and then further processed by gel visualization and gene sequencing (Setianingsih et al., 2019). However, for the diagnosis of COVID-19, PCR is the most recommended test. Microfluidics coupled with PCR (MFQPCR) results can be accelerated, providing fast (up to 1 h) and accurate results (Zhou et al., 2014). However, there are certain limitations to the use of PCR. These include obtaining false-positive results, a long processing time, and low specificity and sensitivity. With quantitative microbial risk assessment, MFQPCR can quantitatively detect various pathogens in freshwater contaminated by waterfowl feces (Ishii et al., 2014) with high sensitivity. Wastewater contains a large number of pathogenic RNA viruses (Miura et al., 2015; Kobayashi et al., 2017), and microfluidic POCT has an advantage over traditional assays because it employs transportable devices and can carry out tests at diverse sampling sites (Telenti et al., 2021). However, conventional PCR assays are quite rigorous and labor-intensive (Le Guyader et al., 1994; Aw et al., 2009).

#### Real-time PCR

During the pandemic, the most widely used and most favored method for COVID-19 diagnosis was real-time RT-PCR, due to RNA being the genetic material and its several advantages, which significantly helped in the detection of infection in the early phases (Corman et al., 2020). Comparatively, real-time RT-PCR has high specificity and sensitivity compared to general PCR, and the procedure is simple and quantitative (Noh et al., 2017). Considerable efforts have been exerted toward the improvement of the real-time RTPCR method in order to overcome its disadvantages when carrying out the procedure (Basit et al., 2021).

#### Loop-mediated isothermal amplification method

**Figure 2:**
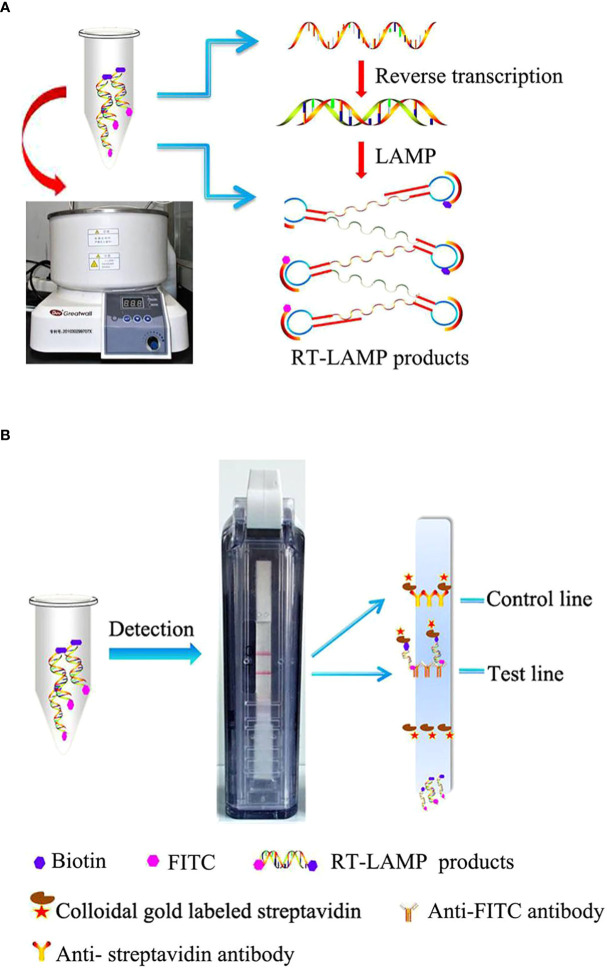
Schematic illustration of the reverse transcription loop-mediated isothermal amplification and vertical flow visualization (RT-LAMP-VF) assay. Figure adopted from Huang et al. (2018). **(A)** RT-LAMP was executed in a water bath at a constant temperature. **(B)** The products of RT-LAMP were detected by using a vertical flow visualization strip.

Among the molecular-based diagnostic methods used for COVID-19, the most prominent during the recent outbreak has been the LAMP method. It has higher sensitivity and specificity rates and amplifies the nucleic acids (DNA/RNA) very rapidly. The method’s procedure involves DNA polymerase and specific primers that synthesize the targeted DNA. The temperature range for this method is 60–65°C, which is a change from that of the ordinary process as it did not denature the strands but only displaced them (Nagamine et al., 2002). At the end point of detection through the LAMP method, the amplified products are further analyzed using gel electrophoresis. Additionally, due to the exponential amplification feature of LAMP, four different primers can detect six different target sequences at the same time (Francois et al., 2011). Within a very short time or a maximum of an hour, up to more than 10^9^ copies of the targeted sequence of loop-form DNA can be amplified by the LAMP assay, with the final product being in the form of many inverted repeats. The use of LAMP for the clinical diagnosis of COVID-19 appears to be extremely useful, reliable, and cost-effective as it does not require expensive instruments or reagents/chemicals. In this case, the *ORF1b* region of SARS-CoV-2 is targeted and amplified by six primers to establish effective viability. A schematic illustration of the combined reverse transcription LAMP and vertical flow visualization (RT-LAMP-VF) assay is shown in **Figure 2**.

#### Enzyme-linked immunosorbent assay

The enzyme-linked immunosorbent assay (ELISA) technique utilizes enzyme immunoassay to detect the ligand in a liquid sample. The antibodies are used against the antigen (Alhajj and Farhana, 2022). This is commonly used in molecular research and diagnostics due to its high reproducibility and specificity, but the traditional ELISA has shown a lot of drawbacks, including difficult procedures, long assay time (4–6 h), and the high volume of reagent used (100 μl). These limitations affect its application in clinical diagnosis (Tan et al., 2017). However, these downsides can be overcome by combining ELISA with microfluidic technology (microfluidic-assisted ELISA). The sample volume can be reduced by almost 20-fold with the capillary force, which pulls the reagent into the reaction chamber during the immunoassay. This results in an overall reagent saving of 5- to 10-fold (Ghosh et al., 2020). The total assay time can also be lessened by 50%, which reduces the inclusive cost of labor (Thaitrong et al., 2013).

## Coronavirus disease modeling using microfluidics

Microfluidic technology is based on the complexity of multicellular and cellular interactions on a microscopic scale, the reproduction of biochemical forces *via* tissue engineering, and the use of organ-on-a-chip technology (Li et al., 2012). The recreation of the cellular microenvironment is the biggest challenge in tissue engineering for CoV research (Prakash et al., 2012). Devices related to microfluidics have been successfully used so that cell-based virus assays could be developed (Berkenbrock et al., 2020b). Tissue engineering related to microfluidics can assist in elucidating the entry mechanism of CoV and the persistence of infection in cells. Antiviral drug discovery can also be accelerated in this manner (Tatara, 2020).

At present, public health communities around the world have been challenged by the high infection and death rates of the recently emerged COVID-19. Such scenarios require the infection to be diagnosed accurately and rapidly in order to control the COVID-19 epidemic, which is only possible with the use of molecular-based diagnostics coupled with microfluidics. Microfluidics can improve diagnostic methods so that a cost-effective and efficient strategy is developed (Udugama et al., 2020). RT-LAMP is an amplification technique for nucleic acids commonly utilized for clinical samples for the effective detection of SARS-CoV-2 (point-of-care) (Mautner et al., 2020). The field of microfluidics can be an alternative to time-consuming benchtop assays. Microfluidic devices capable of manipulating minute amounts of fluids and extracting information from them have been enabled by microelectronics and micro-electromechanical systems (MEMS) (Manz et al., 1990; Prakash et al., 2012).

Recently, a lot of microdevices have been devised to detect small-sized pathogens such as viruses (Yang and Yamamoto, 2016; Zhu et al., 2020). The WHO has recommended the use of qPCR with the combination of RT-PCR (qRT-PCR) for the detection of COVID-19 (Corman et al., 2020). The LAMP technique has now become a commonly used method for molecular diagnostics in benchtop assays, which are based on somewhat constant temperatures for amplification, i.e., between 60°C and 65°C (Notomi et al., 2000). This is compatible with the technique of reverse transcriptase for SARS-CoV-2 benchtop assay (Hong et al., 2004). The benchtop assay based on RT-LAMP showed similar positive results to the RT-qPCR-based protocol in the diagnosis of COVID-19, which was approved by the WHO (Lu et al., 2020). This new protocol can be used for home testing, being applied to microdevices with less risk of spreading the virus. Therefore, the LAMP protocol is now considered a simple method with enormous advantages to be used in microfluidic devices for testing (Nguyen et al., 2020).

The devices related to microfluidics utilize serological testing to detect Zika virus (ZIKV), Middle East respiratory syndrome coronavirus (MERS-CoV), and dengue virus (DENV). Hence, it is also a promising approach to detecting SARS-CoV-2 (Berkenbrock et al., 2020a). These devices are much more efficient and have taken advantage of well-established benchtop assays (implemented in macroscopic analysis tools rather than in miniaturized portable devices), which have been adapted to miniaturized lab-on-a-chip versions. One of the reasons for this is the recent concentrated research effort on SARS-CoV-2, which has unveiled its genetic material, proteins, and other molecules that form the virus, as well as the memory antibodies for the disease (Berkenbrock et al., 2020a; Cao et al., 2020; Li et al., 2020; Turoňová et al., 2020).

## Recent advancements

National wastewater surveillance systems were introduced and implemented worldwide following the COVID-19 pandemic to better understand the extent of SARS-CoV-2 infection in communities. The quantitative monitoring of SARS-Cov-2 within raw sewage can be a good indicator of the progression of viral circulation in a population (Bonanno Ferraro et al., 2021). The Centers for Disease Control and Prevention (CDC) has provided many predictions using various models to estimate future deaths from COVID-19 (Wang et al., 2022). Nevertheless, several advanced techniques have been developed so that the population-level spread of the disease can be tracked statistically (Salathe et al., 2012). Some models have revealed predictive analytic expertise for deaths and hospitalizations (Kissler et al., 2020). Various research groups have been working on wastewater surveillance for COVID-19, including sewage monitoring with the SARS2-EWSP (SARS-CoV-2 Early Warning Wastewater Surveillance Platform), Utah State, in New York (Utah DEQ, 2022) and Tempe, Arizona’s COVID-19 Wastewater Dashboard (Tempe, 2021). The study by Peccia et al. concluded that delaying the sample processing of sludge can wear away the potential of this approach (Larsen and Wigginton, 2020). Recent findings from Bangladesh displayed many contamination results in surface water due to failure in fecal sludge management services (Amin et al., 2020). Risks of waterborne infections can increase due to viral contamination by groundwater recharge in rural areas (Organization, W. H and UniCeF, 2010). On the other hand, various wastewater studies from high-income areas with good sanitation systems are also well-documented (Ahmed et al., 2021; Ahmed et al., 2020b; Kumar et al., 2020; Medema et al., 2020; Randazzo et al., 2020; Rimoldi et al., 2020; Wurtzer et al., 2020a). A significant positive correlation was found between new COVID-19 cases and the viral load in wastewater, suggesting the potential value of clinical testing and wastewater data monitoring in cities (Hoar et al., 2022). Nevertheless, there is still inadequate progress made in the use of wastewater surveillance to monitor the trends in COVID-19, regardless of the applications of several techniques (Bivins et al., 2020; Zahedi et al., 2021). A systematic review reported the detection of almost 7,644 (29.2%) positive samples out of a total of 26,197 samples collected from 66 studies (Shah et al., 2022). Similarly, there are reports of wastewater surveillance done in different settings, including colleges, hospitals, nursing homes, and dormitories (Gonçalves et al., 2022). Reports from the USA showed high consistency between the results of clinical testing of COVID-19 and wastewater samples collected from college residents (Colosi et al., 2021). The viral RNA detected in wastewater from Arizona University campus was directed for specific clinical testing to isolate infected individuals. Eventually, positive wastewater samples provided an early warning of the presence of infection, preventing possible disease transmission (Betancourt et al., 2021). Another study from a university in the US identified asymptomatic COVID-19 cases that were not detected clinically (Gibas et al., 2021). A lot of infected cases were detected by following the SARS-CoV-2 presence in sewage in nursing places in Spain within a time frame of 5–19 days (Davó et al., 2021). The hospital wastewater in Slovenia was found to contain SARS-CoV-2 RNA with a low prevalence of COVID-19 reported at the time (José Gonçalves et al., 2021). In Spain, the estimation of a high proportion of active spillers from SARS-CoV-2 RNA in wastewater indicated infection in individuals who are asymptomatic (Chavarria-Miró et al., 2021). Moreover, positive signals at a minimum of 33 times within 3 months were detected in New South Wales, in which raw sewage inflowing accounted for most of the positive cases, followed by treated effluents after filtration, primary sludge, and river samples (Shah et al., 2022). High sample positivity was reported by Peccia et al., which resulted in positive detections in almost 17,661 samples (20.6%) from primary sludge (Peccia et al., 2020).

## Discussion

Better management of the pandemic requires the precise and timely identification of individuals with SARS-CoV-2, which relies on using suitable testing in various clinical settings for better clinical decision-making (Daughton, 2020). The RNA level of SARS-CoV-2 in sewage correlated with the prevalence of COVID-19, highlighting the efficacy of sewage surveillance as a monitoring tool (Medema et al., 2020). Although there has been a significantly increase in testing capability in developed countries, obvious disproportions in some countries are still evident, especially in developing regions where there is a lack of testing facilities and clinical infrastructure (Abdullahi et al., 2020; Giri and Rana, 2020). This issue must prompt the WHO to encourage researchers to focus their exertions on developing point-of-care assays for community use (Organization, 2020). Advanced techniques such as isothermal amplification methods (e.g., RT-LAMP) are widely adopted because of their low cost and fast processing time, but a lot of false-positive results have also been reported due to nonspecific amplification. This raises concerns regarding their extensive implementation (Rolando et al., 2020). Modern diagnostics provides core diagnostic solutions to carry out large numbers of tests in a timely manner (Pfefferle et al., 2020). An accurate diagnosis of a disease is vital to containing its spread, as a study has estimated that 80% of positive cases of COVID-19 were originally spread by undetected infections (Peccia et al., 2020). Molecular assays are being developed so that the clinical sensitivity and ease-of-use of diagnostic tests can be enhanced (Chan et al., 2020).

With the official announcement of the COVID-19 pandemic by the WHO on March 11, 2020, millions of lives worldwide have been negatively affected by the virus (Franchi, 2020). The developmental timelines of the COVID-19 vaccines were quite long (Corey et al., 2020), along with the risk of SARS-CoV-2 immunity declining with time (Kissler et al., 2020). Therefore, a powerful, authentic, and simultaneous indicator can assist in timely interventions for public health in the case of outbreaks. Various tracking measures have been used for COVID-19, including death rates, confirmed cases, and hospitalizations. However, inefficiencies were discovered in the distribution processes, assembly, and data collection, including reporting delays (Lipsitch and Santillana, 2019). As an acknowledgment to fill such gaps, several recent works have revolved around the potential use of WBE corresponding to clinical testing (Thompson et al., 2020; Venugopal et al., 2020). WBE has been successfully used to track various uses of drugs and other factors. Recently, its popularity has increased in water-related areas with its practical implications *via* its ability to identify underreported and asymptomatic patients with infectious diseases (Sims and Kasprzyk-Hordern, 2020). The LAMP protocol is now considered a simple method with enormous advantages for use in microfluidic devices for testing. Combined with reverse transcription, it can be a better option for the monitoring of COVID-19 infection through WBE. Nevertheless, its practical applications in the surveillance of COVID-19 have only been associated with clinical testing capacity (Amoah et al., 2021). More research is needed to address its wide application.

Currently, a wide range of clinical examinations and molecular diagnostics for suspected cases can reduce the occurrence of false-negative results; moreover, a detailed and careful examination of the commercial tests must be conducted to identify errors and regulate the efficacy of the approved tests (Sharfstein et al., 2020). These would help in better understanding COVID-19 diagnostics to improve the monitoring of emerging infectious diseases in the future. The development of better laboratory assays should not delay the effort to develop rapid diagnostic tests, even with the low precision during the early phase of the outbreak, as rapid tests can play a significant role in the fight against infection in the initial days while highly efficient assays are in the development stages (Ramdas et al., 2020).

## Future remarks

Independent assessments of emerging technologies can provide alternative diagnostic solutions with faster and better screening of individuals with suspected infection. The implementation of targeted, timely, and appropriate testing to reduce the effects of the epidemic is necessary. Generally, epidemic testing is hampered by the lack of options other than test applications for clinical diagnosis and the detection of infections. The wastewater surveillance approach needs to frequently increase the number of populations tested until the key level of detection of positive cases is achieved in order to directly indicate the infection spread in a community. Hence, more rigorous testing is needed to increase the ratio significantly. Nevertheless, diagnostic tests were never intended for mass surveillance. These tests are not only time-consuming and costly, but they can also pose serious exposure risks to those who administer them. Alternatives should be used to solve the problem of diagnostic tests being costly and time-consuming by increasing the quantity of conventional diagnostic tests and minimizing the number of tests mandatory to determine a positive case. Utilizing WBE as an early warning system can reduce the high demand for diagnostic testing by supplementing shortages in the supply chain due to limited manufacturing capacity. The usefulness of WBE can be extended by targeting endogenous biomarkers that were significantly increased during the disease outbreak. However, more research is needed with regard to virus detection in wastewater corresponding to its transmission by highlighting diverse geographic areas and collections from wastewater facilities. A comparison of sludge and inflow from the same wastewater plants can help in determining which approach is more sensitive to decrease the case numbers in a population.

## Conclusion

Viral surveillance can be an impartial method for evaluating the viral spread in different regions, even when there is a limitation of resources and diagnosis. These surveillance strategies can be used as “early warning” systems. Studies have pointed out the need for additional strategies and molecular-based assays to validate the presence of enveloped viruses in water, which will increase the accuracy of wastewater surveillance.

## Author contributions

SM and lH wrote the original draft, GY, AMA, SS, and SC contributed to revision,and fund acquisition. All the authors read the manuscript and participated in the review, editing and agreed to the published version of the manuscript.
